# Descriptive review and comparison of clinical outcomes of AFAP patients

**DOI:** 10.1186/1897-4287-9-S1-P25

**Published:** 2011-03-10

**Authors:** Heidi McCoy, Wendy Kohlmann, Deborah W Neklason, Randall W Burt, Ken M Boucher

**Affiliations:** 1 Departments of Medicine, Human Genetics, and Oncological Sciences, University of Utah, Salt Lake City, Utah, USA

## Objective

This study aimed to examine the colonic polyp phenotype, the surgical outcomes, and the reasons for colectomy in individuals with Attenuated Familial Adenomatous Polyposis (AFAP).

## Methods

Colonoscopy and colectomy medical records were obtained for 197 individuals with a known genetic mutation in the region of the *APC* gene causative of AFAP.

## Results

**Figure 1:**
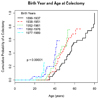
**Age versus cumulative probability of having a colectomy by birth year.** Individuals were divided into five nearly equal cohorts based on birth year.

The number of adenomas was highly variable for both individuals being screened by colonoscopy and those having had a colectomy. The probability of an AFAP patient in this cohort having a colectomy is only 20% at age 40, however, after this age, the probability climbs dramatically. The average age of colectomy is 52 years. By age 70, the cumulative probability of having a colectomy is approximately 80% in this AFAP population. When the population was broken into 5 equal sized birth cohorts (Figure1), we see a trend whereby colectomies are being performed at younger ages in the most recent cohorts (p=0.0001). The major reason for colectomy is a high number or polyps (Figure 1).

## Discussion

Colectomy appears to becoming more common in patients with AFAP and is being recommended at younger ages. A greater number of colectomies were performed in the 1990s which coincided with the identification of the *APC* gene mutation in this family. Subtotal colectomy with ileorectal anastomosis (IRA) is the most common type of colectomy, though 23% still had proctocolectomies with ileoanal anastomosis. Cancer risk did not necessarily correlate with polyp number as 7 individuals with fewer than 20 polyps developed cancer. In the end several factors should be considered in developing a management plan for individuals with AFAP, including lifestyle, polyp number, comorbidities, adherence to screening and patient attitudes.

